# Intrahepatic intraductal papillary neoplasm of the bile duct with low-grade dysplasia presenting as high biliary obstruction and hepatolithiasis: a case report

**DOI:** 10.3389/fmed.2026.1916026

**Published:** 2026-10-16

**Authors:** Jiankai Long, Jingnan Xue, Nanzi Xie, Longhao Zhang, Kai Zhou

**Affiliations:** 1 Department of Hepatobiliary Surgery, People’s Hospital of Anshun City, Guizhou, Anshun, China; 2 Department of Pathology, People’s Hospital of Anshun City, Guizhou, Anshun, China; 3 Department of Liver Surgery, Peking Union Medical College Hospital, Chinese Academy of Medical Sciences & Peking Union Medical College, Beijing, China

**Keywords:** hepatectomy, hepatolithiasis, intraductal papillary neoplasm of the bile duct, intrahepatic bile duct, IPNB

## Abstract

**Background:**

Intraductal papillary neoplasm of the bile duct (IPNB) is an uncommon biliary epithelial neoplasm and an important precursor lesion of cholangiocarcinoma. The educational value of this case lies in demonstrating that IPNB may be clinically masked by hepatolithiasis and biliary obstruction, creating a significant diagnostic pitfall in routine practice.

**Case presentation:**

A 63-year-old woman presented with recurrent upper abdominal pain for more than 1 year, which had worsened over the preceding 10 days. Routine laboratory tests were unremarkable except for a mildly elevated carbohydrate antigen 19-9 level of 39.8 U/mL. Contrast-enhanced CT demonstrated a focal intraductal lesion in the left intrahepatic biliary system with adjacent cystic or aneurysmal dilatation of the affected bile duct, producing the appearance of localized high biliary obstruction. MRI and MRCP further confirmed marked unilateral dilatation of the left intrahepatic bile ducts with focal obstructive change and associated intraductal abnormality. After comprehensive preoperative evaluation, the patient underwent open left hepatectomy. Intraoperatively, a markedly dilated bile duct containing intraductal papillary adenomatous tissue and gelatinous material was identified. Histopathological examination confirmed intrahepatic IPNB with low-grade dysplasia, pancreatobiliary type. No invasive carcinoma was identified, and all resection margins were negative. Immunohistochemistry showed positive staining for CK19, CK7, MUC1, MUC5AC, and MUC6, with negative staining for MUC2. The postoperative course was uneventful, and the patient was discharged on postoperative day 10. No recurrence or major complication was observed during 2 years of follow-up.

**Conclusion:**

In patients with hepatolithiasis and recurrent biliary symptoms, unilateral or focal intrahepatic duct dilatation and localized obstructive biliary abnormalities should prompt consideration of IPNB rather than uncomplicated stone disease alone. Early recognition, definitive surgical resection for localized disease, and complete pathological evaluation are essential for accurate diagnosis, risk stratification, and follow-up planning.

## 1 Introduction

Intraductal papillary neoplasm of the bile duct (IPNB) is an uncommon biliary epithelial neoplasm characterized by papillary or villous proliferation within the bile duct lumen, often accompanied by bile duct dilatation and mucin hypersecretion (1, 2). It is increasingly recognized as an important precursor lesion of cholangiocarcinoma, and its histological grade, invasive status, and subtype are closely associated with prognosis and management strategies (3). However, preoperative recognition of IPNB remains difficult in routine clinical practice because its symptoms are often nonspecific and its imaging findings may overlap with those of hepatolithiasis, cholangitis, and intrahepatic cholangiocarcinoma (4).

Although IPNB associated with hepatolithiasis has been reported previously, its diagnosis may still be delayed or overlooked when biliary stones appear sufficient to explain recurrent symptoms and ductal obstruction (5). In such settings, the main clinical challenge is not simply the rarity of IPNB itself, but the risk of misinterpreting a true intraductal neoplasm as uncomplicated stone-related biliary disease. Practical recognition of this diagnostic pitfall is important because focal or unilateral bile duct dilatation, localized obstructive change, intraductal soft-tissue components, or abundant mucin may indicate IPNB rather than hepatolithiasis alone (6, 7).

Here, we report a case of intrahepatic IPNB with low-grade dysplasia, pancreatobiliary type, in a 63-year-old woman presenting with recurrent upper abdominal pain, hepatolithiasis, and high biliary obstruction. The educational value of this case lies in demonstrating how IPNB can be clinically masked by hepatolithiasis and obstructive biliary changes, making preoperative diagnosis challenging. This report further highlights the importance of careful radiologic assessment, timely surgical resection for localized disease, and complete pathological evaluation, including subtype classification, margin assessment, and exclusion of invasive carcinoma, in order to achieve accurate diagnosis and appropriate follow-up planning (6–8).

## 2 Case presentation

A 63-year-old woman was admitted to our hospital with recurrent upper abdominal pain for more than 1 year, with worsening symptoms over the preceding 10 days. The pain was intermittent and distending in nature, mainly located in the right upper abdomen. She had no nausea, vomiting, chills, high fever, jaundice, or diarrhea. She had previously received symptomatic treatment at another hospital for presumed gallstones, with temporary relief. Her past medical history, medication history, allergy history, family history, and social history were unremarkable according to the available records. Her main concerns were symptom relief, clarification of the diagnosis, and definitive treatment.

On admission, no remarkable positive findings were documented on physical examination. Laboratory investigations, including complete blood count, liver and renal function tests, and coagulation profile, were within normal limits. The carbohydrate antigen 19-9 level was mildly elevated at 39.8 U/mL.

Preoperative imaging suggested a localized left-sided biliary lesion rather than uncomplicated hepatolithiasis alone. As shown in Figure 1, contrast-enhanced CT demonstrated a focal intraductal adenomatous lesion in the left intrahepatic biliary system with adjacent cystic or aneurysmal dilatation of the affected bile duct, producing the appearance of localized high biliary obstruction. The lesion was centered near the left hepatic hilar region and was associated with unilateral left intrahepatic biliary dilatation. MRI and MRCP further confirmed marked unilateral dilatation of the left intrahepatic bile ducts with focal hilar-level obstructive change and associated intraductal abnormality (Figure 2). The predominantly unilateral distribution of ductal dilatation and the localized obstructive appearance were atypical for simple hepatolithiasis alone and raised concern for an underlying intraductal biliary neoplasm. Based on the clinical presentation and radiologic findings, the preoperative differential diagnosis included hepatolithiasis with cholangitis, intrahepatic cholangiocarcinoma, mucinous biliary neoplasm, and intraductal papillary neoplasm of the bile duct (IPNB).

**Figure 1:**
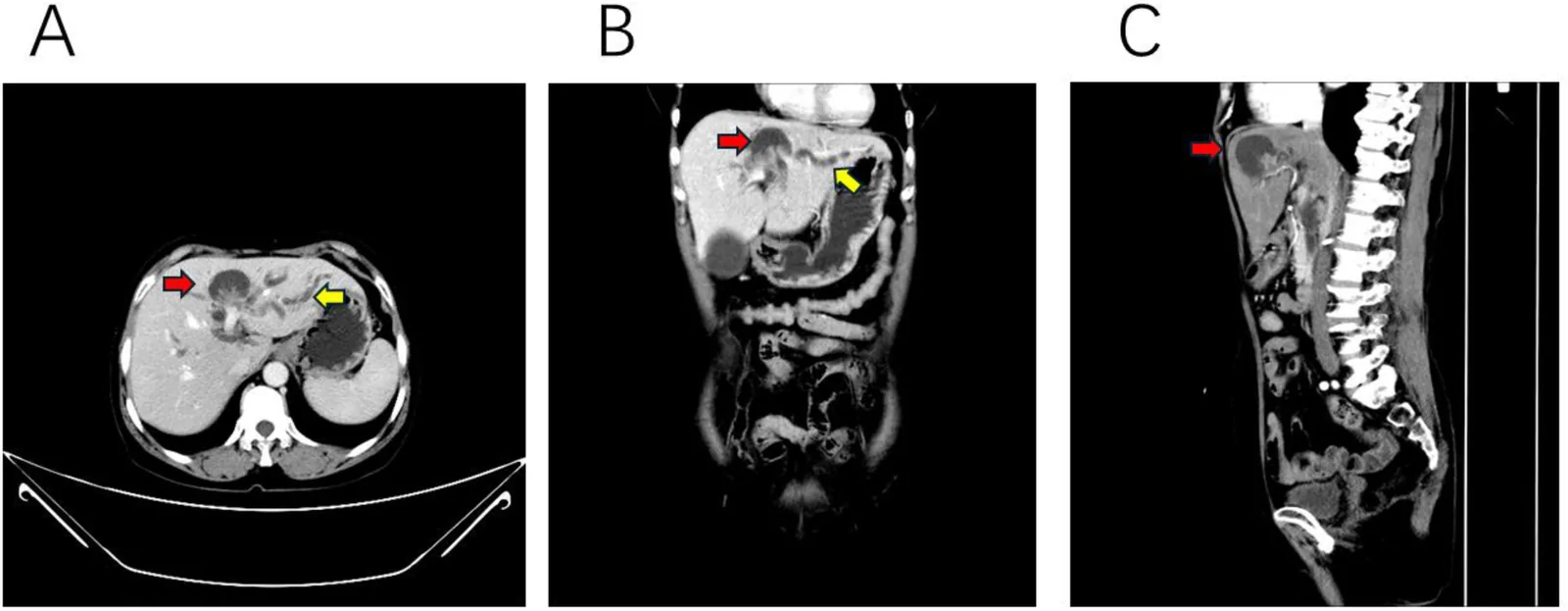
Preoperative CT findings. **(A)** Axial contrast-enhanced CT showing a focal intraductal adenomatous lesion in the left intrahepatic biliary system (red arrow) and associated dilatation of the adjacent bile duct (yellow arrow). **(B)** Coronal contrast-enhanced CT demonstrating localized high biliary obstruction in the left hepatic lobe, with the intraductal adenomatous lesion indicated by the red arrow and the dilated bile duct indicated by the yellow arrow. **(C)** Sagittal contrast-enhanced CT further showing focal left-sided biliary obstruction and cystic/aneurysmal dilatation of the affected intrahepatic bile duct (red arrow, adenomatous lesion).

**Figure 2:**
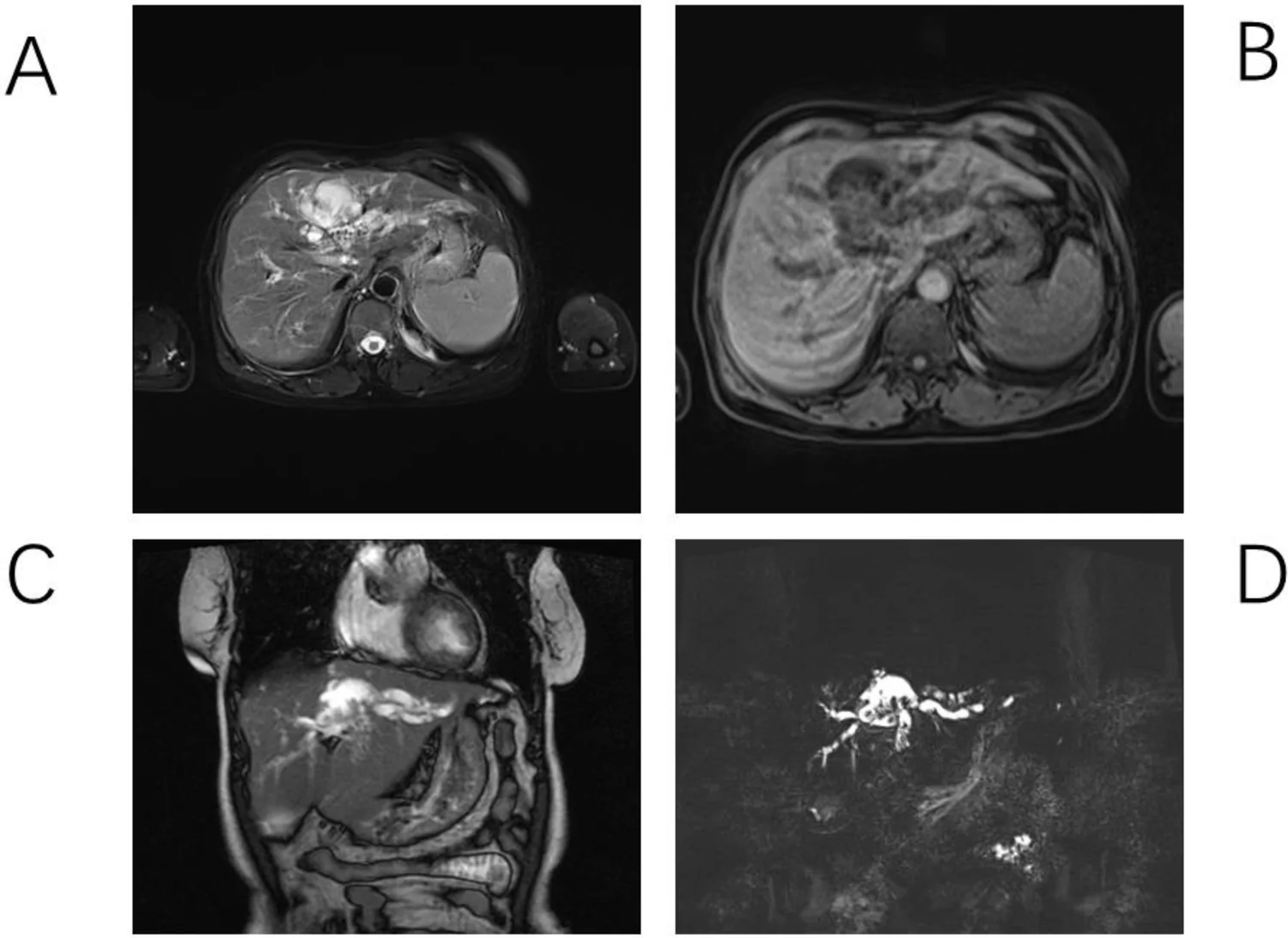
Preoperative MRI and MRCP findings. **(A,B)** Axial MRI showing focal abnormality in the left intrahepatic biliary system with marked dilatation of the adjacent bile ducts. **(C)** Coronal MRI demonstrating unilateral left intrahepatic duct ectasia. **(D)** MRCP showing marked dilatation of the left intrahepatic bile ducts with focal obstructive change, corresponding to localized intraductal biliary disease.

Given that the lesion appeared to be localized to the left intrahepatic biliary system and that the patient had recurrent obstructive symptoms, surgical treatment was selected after comprehensive preoperative evaluation. The patient underwent open left hepatectomy under general anesthesia. Intermittent inflow occlusion using the Pringle maneuver was applied, with a total clamping time of 30 min. Intraoperative blood loss was approximately 20 mL, and the patient remained hemodynamically stable throughout the procedure. The intraoperative and gross specimen findings are shown in Figure 3. Gross examination of the resected specimen demonstrated a markedly dilated bile duct containing intraductal papillary adenomatous tissue and gelatinous material.

**Figure 3:**
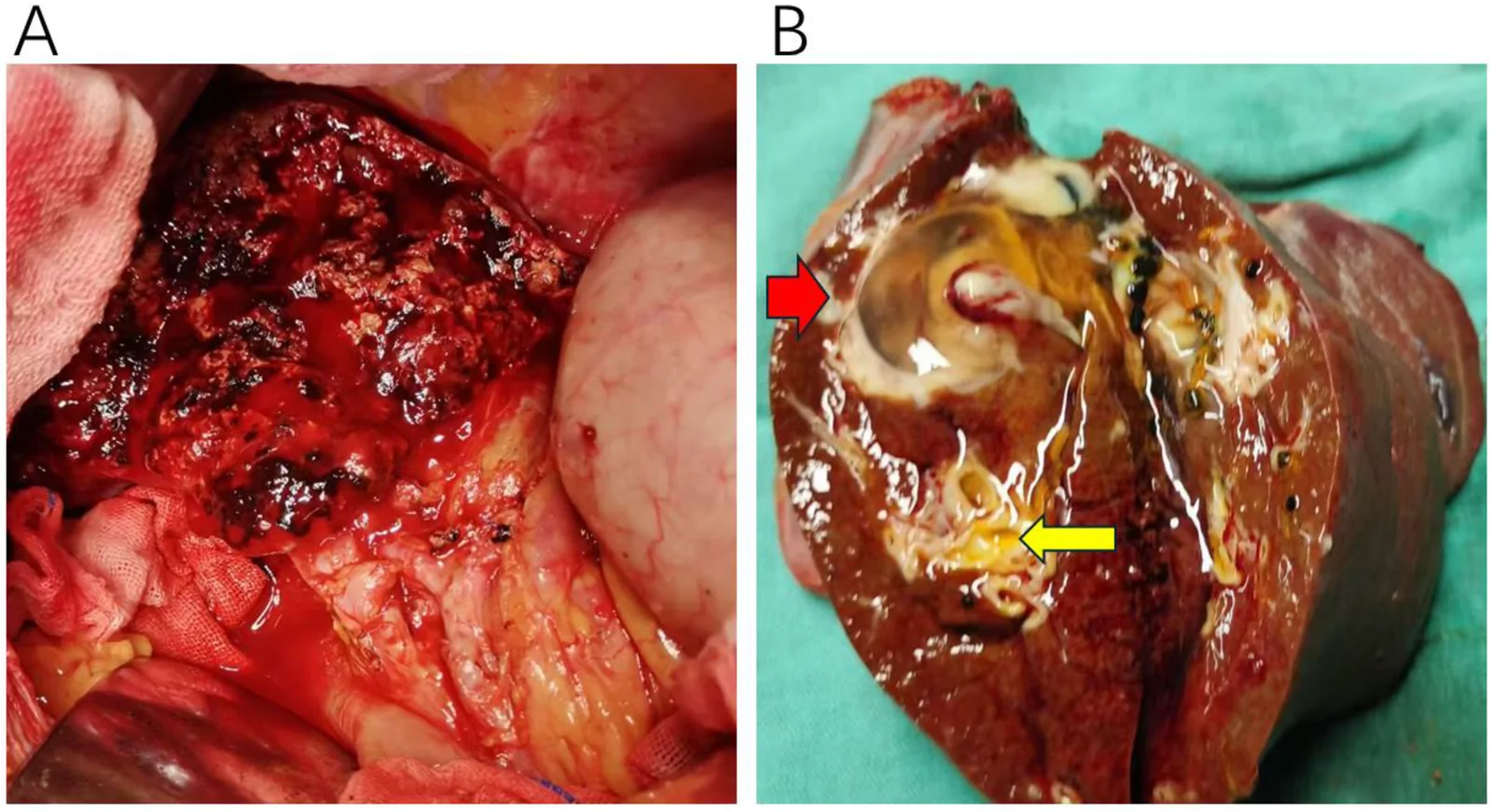
Intraoperative and gross specimen findings. **(A)** Intraoperative view during open left hepatectomy. **(B)** Gross examination of the resected specimen showing a markedly dilated bile duct (yellow arrow) containing intraductal papillary adenomatous tissue (red arrow) and gelatinous/mucinous material.

Representative histopathological and immunohistochemical findings are shown in Figure 4. Histopathological examination confirmed intrahepatic intraductal papillary neoplasm of the bile duct with low-grade dysplasia, pancreatobiliary type. No invasive carcinoma was identified, and all resection margins were negative. Immunohistochemical staining showed that the neoplastic biliary epithelium was positive for CK19, CK7, MUC1, MUC5AC, and MUC6, but negative for MUC2, further supporting the diagnosis and subtype classification.

**Figure 4:**
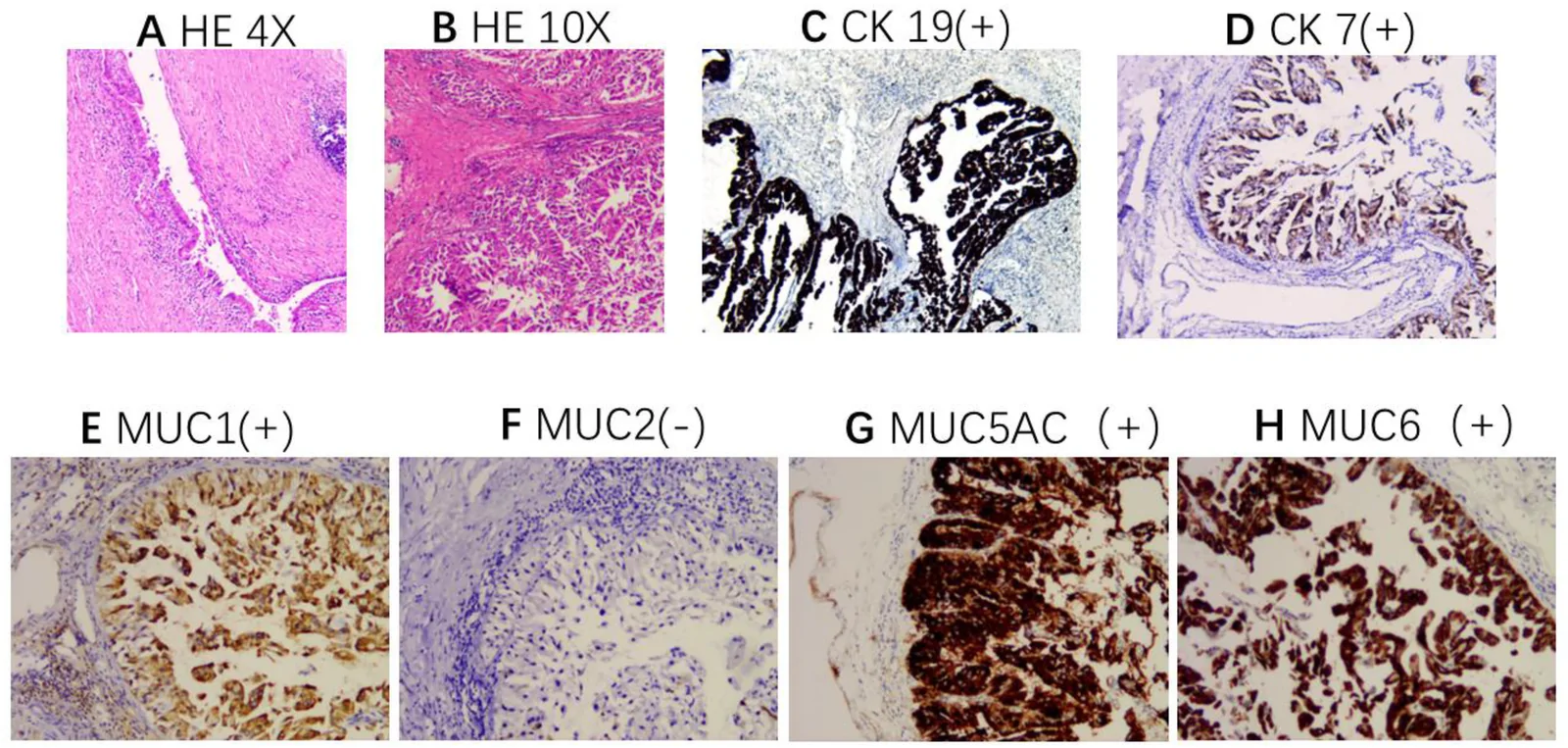
Histopathological and immunohistochemical findings of intrahepatic intraductal papillary neoplasm of the bile duct (IPNB). **(A)** Low-power hematoxylin and eosin staining (HE, ×4) showing papillary epithelial proliferation within the bile duct lumen and its relationship to the bile duct wall. **(B)** Medium-power hematoxylin and eosin staining (HE, ×10) showing complex papillary and glandular epithelial proliferation within the lesion. **(C,D)** Immunohistochemical staining showing positivity for CK19 and CK7 in the neoplastic biliary epithelium. **(E–H)** Immunohistochemical staining showing positivity for MUC1, MUC5AC, and MUC6, with negativity for MUC2, supporting the diagnosis of pancreatobiliary-type IPNB.

The postoperative course was uneventful, and the patient was discharged on postoperative day 10 without documented complications. During 2 years of outpatient follow-up, the patient remained clinically stable, with relief of abdominal symptoms after surgery. The latest CT demonstrated postoperative changes after left hepatectomy, possible intrahepatic bile duct calculi or calcifications, mild intrahepatic and extrahepatic bile duct dilatation, and a right renal cyst. No definite recurrent lesion was reported.

## 3 Timeline

Table 1.

**Table 1:** Timeline of the case.

Date	Event	Key findings
May 2020	Onset of symptoms	Intermittent upper abdominal pain, mainly in the right upper quadrant, without jaundice or fever
May 2021	Outpatient imaging evaluation	CT and MRI revealed left intrahepatic bile duct dilatation, multiple stones, a left hepatic lesion, and high biliary obstruction
May 2021	Hospital admission and preoperative assessment	Routine laboratory tests were unremarkable; CA19-9 was mildly elevated at 39.8 U/mL
May 2021	Surgical treatment	Open left hepatectomy was performed under general anesthesia; intermittent Pringle maneuver for 30 min; blood loss approximately 20 mL
May 2021	Intraoperative findings	Multiple fragile gray-yellow papillary lesions and gelatinous material were found within the bile duct lumen
Postoperative day 10	Discharge	Uneventful recovery without major complications
June 2021 to May 2023	Follow-up	No recurrence or major complications were observed during 2 years of follow-up

## 4 Diagnostic assessment

The patient presented with recurrent upper abdominal pain for more than 1 year, with worsening symptoms over the preceding 10 days. Laboratory investigations, including complete blood count, liver and renal function tests, and coagulation profile, were within normal limits. Serum carbohydrate antigen 19-9 was mildly elevated at 39.8 U/mL.

Preoperative imaging suggested a localized left-sided biliary lesion rather than uncomplicated hepatolithiasis alone. As shown in Figure 1, contrast-enhanced CT demonstrated a focal intraductal adenomatous lesion in the left intrahepatic biliary system with adjacent cystic or aneurysmal dilatation of the affected bile duct, producing the appearance of localized high biliary obstruction. MRI and MRCP further confirmed marked unilateral dilatation of the left intrahepatic bile ducts with focal hilar-level obstructive change and associated intraductal abnormality (Figure 2). The predominantly unilateral distribution of ductal dilatation and the localized obstructive appearance were atypical for simple hepatolithiasis alone and raised concern for an underlying intraductal biliary neoplasm.

Based on the clinical presentation and radiologic findings, the preoperative differential diagnosis included hepatolithiasis with cholangitis, intrahepatic cholangiocarcinoma, mucinous biliary neoplasm, and intraductal papillary neoplasm of the bile duct (IPNB). Definitive diagnosis was established by postoperative histopathological examination, which confirmed intrahepatic IPNB with low-grade dysplasia, pancreatobiliary type. No invasive carcinoma was identified, and all resection margins were negative. Immunohistochemical staining showed positivity for CK19, CK7, MUC1, MUC5AC, and MUC6, with negative staining for MUC2, further supporting the diagnosis and subtype classification (Figure 4).

## 5 Therapeutic intervention

Considering that the lesion was localized to the left intrahepatic biliary system and that the patient had recurrent symptoms suggestive of obstructive biliary disease, surgical treatment was selected after comprehensive preoperative evaluation.

The patient underwent open left hepatectomy under general anesthesia. Intermittent inflow occlusion using the Pringle maneuver was applied, with a total clamping time of 30 min. Intraoperative blood loss was approximately 20 mL, and the patient remained hemodynamically stable throughout the procedure. The intraoperative and gross specimen findings are shown in Figure 3. Gross examination of the resected specimen demonstrated a markedly dilated bile duct containing intraductal papillary adenomatous tissue and gelatinous/mucinous material.

No additional intraoperative adverse events were documented.

## 6 Follow-up and outcomes

The postoperative course was uneventful. No major complications, such as bile leakage, hemorrhage, or severe infection, were documented during hospitalization. The patient recovered well and was discharged on postoperative day 10.

Histopathological examination of the surgical specimen confirmed intrahepatic intraductal papillary neoplasm of the bile duct with low-grade dysplasia, pancreatobiliary type. No invasive carcinoma was identified, and all resection margins were negative. Immunohistochemical findings supported the diagnosis, showing positivity for CK19, CK7, MUC1, MUC5AC, and MUC6, with negativity for MUC2.

During 2 years of outpatient follow-up, the patient remained clinically stable, with no evidence of recurrence or major postoperative complications. Her abdominal symptoms resolved after surgery, and her quality of life improved.

## 7 Discussion

IPNB is an uncommon biliary epithelial neoplasm characterized by intraductal papillary growth, often accompanied by bile duct dilatation and mucin production, and is increasingly recognized as an important precursor lesion of cholangiocarcinoma (9). However, the main clinical challenge is not simply its rarity, but the fact that IPNB may be overlooked when coexisting hepatolithiasis and biliary obstruction appear sufficient to explain the patient's symptoms and imaging abnormalities (10).

The educational value of the present case lies in this diagnostic pitfall. The patient presented with recurrent biliary symptoms and multiple intrahepatic stones, a scenario that could easily be interpreted as uncomplicated hepatolithiasis with secondary obstruction. However, preoperative imaging showed unilateral left intrahepatic bile duct dilatation with focal high biliary obstruction and an associated intraductal lesion, rather than diffuse stone-related biliary disease alone (Figures 1, 2). This pattern should prompt suspicion for an intraductal neoplasm, particularly when the distribution of ductal dilatation is focal or unilateral and appears disproportionate to the stone burden (10, 11).

Radiologic-pathologic correlation was particularly informative in this case. The focal intraductal lesion and adjacent dilated bile duct seen on CT (Figure 1) corresponded to the gross finding of papillary adenomatous tissue within a markedly dilated bile duct containing gelatinous material (Figure 3B) and to the microscopic finding of papillary epithelial proliferation on histopathology (Figures 4A,B). This correlation helps explain why the lesion was not merely a consequence of stone disease, but represented a true intraductal neoplasm.

For lesions confined to one intrahepatic biliary system, hepatectomy can provide both definitive diagnosis and curative treatment. In the present case, open left hepatectomy resulted in an uneventful postoperative recovery and a favorable 2-year outcome without documented recurrence. These findings support timely surgical resection when localized intraductal biliary neoplasia is suspected and technically resectable.

Pathological evaluation is essential not only for diagnosis but also for clinically meaningful classification. In this case, histopathological examination confirmed low-grade pancreatobiliary-type IPNB, with negative resection margins and no invasive carcinoma. Immunohistochemical staining demonstrated positivity for CK19, CK7, MUC1, MUC5AC, and MUC6, with negativity for MUC2 (Figures 4C–H), supporting pancreatobiliary differentiation. These findings strengthen the diagnostic classification and provide more meaningful comparison with previously reported cases than morphology alone.

Even when the lesion is low grade and completely resected, long-term surveillance remains necessary because biliary neoplasms may recur or present as multifocal disease (12). From a practical perspective, patients with IPNB should undergo periodic clinical review and hepatobiliary imaging after surgery, especially during the first 2 postoperative years.

His report has several limitations. First, several detailed preoperative radiologic parameters, including reproducible lesion measurements, formal enhancement characteristics, and complete archived MRCP descriptors, were unavailable. Second, as a single-case report, the generalizability of the findings is inherently limited. Finally, although the patient remained clinically stable without definite evidence of recurrence during the 2-year follow-up period, this duration may be insufficient to exclude late recurrence or the development of multifocal disease, both of which remain possible in IPNB. Therefore, continued long-term clinical and imaging surveillance is warranted.

Overall, this case emphasizes that in patients with hepatolithiasis and recurrent biliary symptoms, unilateral or focal intrahepatic duct dilatation, localized obstructive change, and intraductal abnormalities should raise suspicion for IPNB rather than uncomplicated stone disease alone. Early recognition, appropriate surgical resection, and complete pathological evaluation are essential for accurate diagnosis, risk stratification, and follow-up planning.

## 8 Conclusion

Intrahepatic IPNB with low-grade dysplasia may be clinically masked by hepatolithiasis and biliary obstruction, making preoperative diagnosis difficult. In patients with recurrent biliary symptoms, unilateral or focal intrahepatic duct dilatation, localized obstructive change, and intraductal abnormalities should prompt consideration of IPNB rather than uncomplicated stone disease alone. In the present case, surgical resection enabled definitive diagnosis and treatment, and pathological evaluation confirmed low-grade pancreatobiliary-type IPNB with negative resection margins and no invasive carcinoma. Early recognition, timely surgery for localized disease, and complete pathological assessment are essential for accurate diagnosis, risk stratification, and follow-up planning.

## 9 Patient perspective

After surgery, the patient reported that her abdominal pain had resolved and that follow-up examinations were reassuring. She expressed satisfaction with the treatment outcome and felt more secure in her daily life.

## Data Availability

The original contributions presented in the study are included in the article/Supplementary Material, further inquiries can be directed to the corresponding author.

## References

[1] Dias NG Mendes JDT Kozlowski BA Parente DB Martins DL Nunes TF. Intraductal papillary neoplasm of the bile duct: simplifying findings. Semin Ultrasound CT MR. 2025,46(6):415–27. 10.1053/j.sult.2025.09.005 40935089

[2] Aoki Y Mizuma M Hata T Aoki T Omori Y Ono Y. Intraductal papillary neoplasms of the bile duct consist of two distinct types specifically associated with clinicopathological features and molecular phenotypes. J Pathol. (2020) 251(1):38–48. 10.1002/path.5398 32100878

[3] Kim KM Lee JK Shin JU Lee KH Lee KT Sung J-Y. Clinicopathologic features of intraductal papillary neoplasm of the bile duct according to histologic subtype. Am J Gastroenterol. (2012) 107(1):118–25. 10.1038/ajg.2011.316 21946282

[4] Park HJ Kim SY Kim HJ Lee SS Hong GS Byun JH. Intraductal papillary neoplasm of the bile duct: clinical, imaging, and pathologic features. AJR Am J Roentgenol. (2018) 211(1):67–75. 10.2214/AJR.17.19261 29629808

[5] Aslam A Wasnik AP Shi J Sahai V Mendiratta-Lala M. Intraductal papillary neoplasm of the bile duct (IPNB): CT and MRI appearance with radiology-pathology correlation. Clin Imaging. (2020) 66:10–7. 10.1016/j.clinimag.2020.04.036 32438236 PMC7577174

[6] Sakai Y Ohtsuka M Sugiyama H Mikata R Yasui S Ohno I. Current status of diagnosis and therapy for intraductal papillary neoplasm of the bile duct. World J Gastroenterol. (2021) 27(15):1569–77. 10.3748/wjg.v27.i15.1569 33958844 PMC8058653

[7] Mocchegiani F Vincenzi P Conte G Nicolini D Rossi R Cacciaguerra AB. Intraductal papillary neoplasm of the bile duct: the new frontier of biliary pathology. World J Gastroenterol. (2023) 29(38):5361–73. 10.3748/wjg.v29.i38.5361 37900587 PMC10600795

[8] Lluís N Serradilla-Martín M Achalandabaso M Jehaes F Dasari BVM Mambrilla-Herrero S. Intraductal papillary neoplasms of the bile duct: a European retrospective multicenter observational study (EUR-IPNB study). Int J Surg. (2023) 109(4):760–71. 10.1097/JS9.0000000000000280 PMC10389541 36917142

[9] Zen Y Akita M. Neoplastic progression in intraductal papillary neoplasm of the bile duct. Arch Pathol Lab Med. (2024) 148(9):989–96. 10.5858/arpa.2022-0407-RA 36800543

[10] Huang X Chen T Liu H Huang M. A review of type 1 and type 2 intraductal papillary neoplasms of the bile duct. Curr Med Sci. (2024) 44(3):485–93. 10.1007/s11596-024-2863-5 38748369

[11] Stendahl K Gilani SM Basturk O Hui P Sigel C Cai G. Intraductal papillary neoplasm of the bile duct: cytomorphologic and molecular features. Cancer Cytopathol. (2023) 131(1):37–49. 10.1002/cncy.22637 36041224 PMC9812877

[12] Toti L Manzia TM Di Giuliano F Picchi E Tariciotti L Pedini D. Intraductal papillary neoplasms of the bile duct: clinical case insights and literature review. Clin Pract. (2024) 14(5):1669–81. 10.3390/clinpract14050133 39311283 PMC11417733

